# The effect of timpanoplasty on tinnitus in patients with conductive hearing loss: a six month follow-up

**DOI:** 10.1016/S1808-8694(15)30083-5

**Published:** 2015-10-19

**Authors:** Adriana da Silva Lima, Tanit Ganz Sanchez, Maria Flávia Bonadia Moraes, Silvia Cristina Batezati Alves, Ricardo Ferreira Bento

**Affiliations:** a PhD in Otorhinolaryngology -FMUSP. MD. Otolaryngologist; b Associate Professor of Otolaryngology - FMUSP. Associate Professor of Otolaryngology - USP; c Speech and Hearing therapist - Department of Audiology - Discipline of Otorhinolaryngology - Medical School - University of São Paulo; d PhD student in Otorhinolaryngology - Medical School - USP. Otolaryngologist; e Associate Professor of Otolaryngology - USP. Head of the Otorhinolaryngology and Ophthalmology department - FMUSP. Work carried out at the Tinnitus Research Group - Otorhinolaryngology Clinic - University of São Paulo Medical School

**Keywords:** chronic otitis media, tympanoplasty, tinnitus

## Abstract

Tympanoplasty is done to eradicate ear pathology and to restore the conductive hearing mechanism (eardrum and ossicles). Some patients, however, do not tolerate tinnitus and question physicians about the results of surgery when tinnitus persists. **Aim:** to evaluate the progression of tinnitus in patients with conductive hearing loss after tympanoplasty. **Study Design:** a prospective cohort study. Material and Methods: 23 consecutive patients with tinnitus due to chronic otitis media underwent tympanoplasty. The patients underwent a medical and audiological protocol for tinnitus before and after tympanoplasty. **Results:** 82.6% of patients had improvement or elimination of tinnitus after tympanoplasty The mean score of postoperative intolerance to tinnitus (1.91 for 30 and 180 days) was significantly different from preoperative scores (5.26). As to hearing loss, patients improved medically 30 and 180 days after surgery (3.65 and 2.91) compared to the preoperative condition (6.56). Audiometry revealed improvement at all frequencies from 0.25 to 6KHz, except at 8KHz. The air-bone gap was closed or was within 10dB in 14 cases (61%). An intact tympanic membrane was achieved in 78% of the cases. **Conclusion:** Aside from the classical improvement of hearing loss, tympanoplasty also offers good control of tinnitus.

## INTRODUCTION

Despite recent advances in tinnitus studies, it has been mainly associated with cochlear, auditory nerve or central auditory pathway diseases^1^.

Although tinnitus physiopathology is complex and with many theories already described as a means to justify it, our current idea is that, most of the times, it is a consequence of plastic alterations that happen to central auditory pathways because of a hearing loss caused by a lesion to the periphery pathway. In this case, the hearing loss reversion could oppose such plastic alterations with time and then reduce tinnitus.

Among the diseases that join hearing loss and tinnitus, we mention otospongiosis and middle ear acute or chronic inflammatory processes, such as otitis media with effusion, simple or suppurative chronic otitis, with or without cholesteatoma. Patients with external otitis may also complain of tinnitus associated with pain and edema, although its presence is usually temporary^2^.

In our daily practice, the most common complaints of patients with chronic simple otitis media are constant otorrhea and hearing loss. However, those that also complain of tinnitus may have clinical repercussions, however this is usually not valued enough by health care professionals.

Tympanoplasty is the procedure of choice for the treatment of simple chronic otitis media. Its main goals are to eradicate middle ear disease and restore sound conduction mechanisms, including the tympanic membrane and the ossicles. Its indication is mainly in cases of tympanic perforations without spontaneous recovery and hearing loss. Nonetheless, as we have seen in our tinnitus research group, some patients ask the otolaryngologist, when discussing the surgical option, about the possible impact that such procedure may have on tinnitus. In the literature, the few papers that mention tinnitus are usually restricted to a retrospective analysis on the presence or absence of such symptom in the pre and postoperative, without showing its detailed evolution. Although its association with exclusively conductive hearing loss is very little studied, it is full of relevance because the conductive component is more easily reverted with treatment, when compared with its sensorineural counterpart. Therefore, our goal is to prospectively assess the tympanoplasty effect in the short and middle term on tinnitus and the conductive hearing loss of patients with chronic simple otitis media.

## MATERIALS AND METHODS

This study was approved by the Research Projects Analysis Committee of the University of São Paulo Medical School (CAPPesq), under protocol # 657/02.

### Sample definition

Patient inclusion criteria were:

1. Constant unilateral or bilateral tinnitus;
2. Tonal audiometry showing conductive hearing loss;
3. Diagnosis matching that of chronic simple otitis media;
4. Indication of tympanoplasty in the tinnitus-affected ear;
5. Information on the research and signed informed consent.

In cases of bilateral surgical indication, we considered only data from the operated ear.

We excluded patients with:

1. Previous otologic surgery in the tinnitus ear;
2. Chronic suppurative otitis media with or without cholesteatoma;
3. Need to reconstruct the ossicular chain during surgery;
4. Clinical or audiologic contra-indication to surgery;
5. Postoperative audiogram showing mixed or sensorineural hypoacusis in any frequency.

There were 47 patients initially assessed, however, 24 were taken off the study. Thus, the final sample was made up of 23 patients, 13 women and 10 men. Age varied between 12 and 54 years, with average of 30.52 and median of 30 years.

#### Procedures

All tympanoplasties were carried out between December 2001 and January of 2004, in the Otolaryngology Department of the University of São Paulo Medical School, by different otolaryngology surgeons, usually 2nd year resident physicians under the supervision of a preceptor. Nonetheless, all followed the same surgical technique (medial - underlay), except for one case operated with the overlay technique. Temporal muscle fascia grafts and/or tragal cartilage pericondrium were the most employed methods in the tympanic membrane reconstruction.

Audiologic and medical evaluation protocols were employed before surgery and later on repeated with 30 and 180 days, always by the same professionals (otorhinolaryngologist and audiologist). The discomfort brought about by the tinnitus and the hearing loss to the patient’s life was classified according to a numerical scale from 0 to 10, where 0 means no discomfort and 10 means the maximum discomfort. Scores between 0 and 3 were grouped as mild symptoms, 4 to 7 as moderate and 8 to 10 as severe symptoms, as a routine analysis procedure. We also assessed tonal conduction thresholds in the frequencies of 0.25; 0.5; 1; 2; 4; 6 and 8 KHz and in the bone pathway in the frequencies of 0.5; 1; 2 and 4 KHz before surgery. Tonal threshold was obtained in the frequencies of 0.5; 1; 2 and 4 KHz and the postoperative gap was calculated by subtracting the air conduction tonal threshold from the bone conduction^3^. The graft take rate was evaluated by otoscopy done by the same physician in all the instances.

#### Statistical analysis

We used descriptive analysis tools, just as the Friedman’s non-parametric test, Spearman’s correlation coefficient and the Fisher’s exact test, deemed significant when p < 0.05.

## RESULTS

### Preoperative

#### Tinnitus

As to tinnitus duration before surgery, 13 patients (57%) had had it for less than 5 years, 9 patients (39%) for more than 10 years and only one patient (4%) had had it between 5 and 10 years.

Most of the patients (74%) had moderate to severe discomfort caused by their tinnitus. The remainder (26%) had only mild discomfort. The mean value obtained in the preoperative numeric scale was of 5.26.

#### Hearing loss

We have noticed that 87% of the patients had moderated to severe discomfort related to their hearing loss. Only 13% of the patients had mild discomfort concerning their hearing impairment. The mean value attained was of 6.56, according to the numeric scale.

All the patients had conductive hearing loss, according to the exclusion criteria. The average of air conduction thresholds in the isolated frequencies was of 55 (0.25 KHz); 43.91 0.5 KHz); 35.87 (1 KHz); 33.70 (2 KHz); 38.04 (4 KHz); 41.30 (6 KHz) and 42.17 (8 KHz). Grouping the frequencies in low (0.25 and 0.5KHz), intermediate (1 and 2KHz) and high (4, 6 and 8KHz), we observed the following values: 49.46 in low, 34.78 in intermediate and 40.50 in high frequencies.

### Postoperative

#### Tinnitus

**Graph 1:**
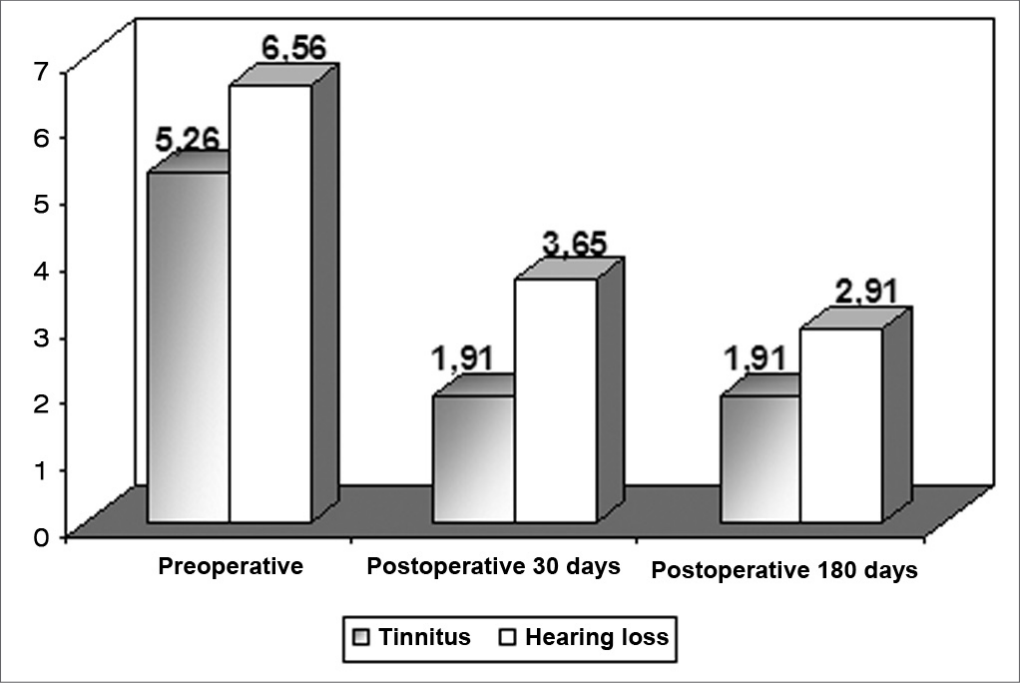
Tinnitus and hearing loss discomfort mean value assessment in the patient’s life before and after tympanoplasty.

In tinnitus subjective analysis after tympanoplasty, we observed that 8 patients (34.8%) had full remission, 11 patients (47.8%) had some improvement, 3 patients (13%) remained with their tinnitus unaltered; and one reported symptoms worsening (4.3%). The average value of tinnitus discomfort was of 1.91 in the 30 and 180 days of postoperative (p<0.001). There were no differences at the 30 and 180 days (Graph 1).

#### Hearing loss

The mean values for hearing loss discomfort were 3.65 and 2.91 in the 30 and 180 days of postoperative, respectively (p<0.001). Times 30 and 180 days did not present significant differences between themselves (Graph 1).

The air conduction threshold averages in the isolated frequencies after 30 and 180 days were, respectively: 31.52dB HL and 33.26dB HL (0.25 KHz), 24.57dB HL and 25.87dB HL (0.5 KHz), 24.35dB HL and 21.30dB HL (1KHz), 22.17dB HL and 19.57dB HL (2KHz), 25dB HL and 21.09dB HL (4KHz), 34.13dB HL and 31.09dB HL (6KHz) and 39.13dB HL and 34.57dB HL (8KHz). All air conduction frequencies from 0.25 to 4 KHz presented significant improvement (p<0.001) with 30 and 180 days in relation to preoperative evaluation. There were no differences between times 30 and 180 days. In regards of the 6 KHz frequency, there was a significant difference only between the pre and postoperative values at 180 days (p<0.05). There were no significant alterations at 8 KHz along the evaluations (p=0.145).

The frequencies grouped in low, middle range and high after 30 and 180 days were: 28.04dB HL and 29.57dB HL (low), 23.26dB HL and 20.43dB HL (middle range) and 32.75dB HL and 28.91dB HL (high). These results were significant in relation to preoperative values (p<0.05). There were no differences in results between 30 and 180 days.

As to the hearing gap closure, we observed that 57% ended up with a gap less than or equal to 10dB in the 30 day postoperative period and 61% at 180 days (Table 1). There was a significant air-bone gap improvement in the postoperative at 30 days (16.90 dB HL, p < 0.05) and 180 days (16.73 dB HL, p < 0.05) in relation to the preoperative time (29.58 dBHL). Results between 30 and 180 days are not different among themselves.

**Table 1:** Air-bone gap in the preoperative and after 30 and 180 days.

GAP	Preop	Postop 30d	Postop 180d
0 a 10dB	0 (0%)	13 (57%)	14 (61%)
11 a 20dB	5 (22%)	6 (26%)	5 (22%)
21 a 30dB	12 (52%)	3 (13%)	1 (4%)
>30dB	6 (26%)	1 (4%)	3 (13%)

TOTAL	23 (100%)	23 (100%)	23 (100%)

**dB:** decibel

Correlation between tinnitus improvement and tonal threshold improvement in the low, intermediate and high frequencies

According to Spearman’s coefficient, there was no correlation between an improvement in tonal thresholds in low, intermediate and high frequencies and tinnitus improvement.

Association between gap closure and tinnitus positive outcomes (improvement or remission) and negative (unaltered or worsening) outcomes.

After 30 days of postoperative, among the 13 patients who obtained a gap between 0-10dB HL, 11 had positive tinnitus responses and 2 had negative responses.

After 180 days of postoperative, among the 14 patients that had gaps between 0-10dB HL, 11 had positive tinnitus responses and 3 had negative responses. Among the 3 patients who remained with a > 30dB HL gap, all had positive tinnitus response. There was no statistical significance in the Fisher’s test among the preoperative results and after 30 and 180 days (p=0.806 and 0.202).

Association between graft take and positive responses (remission or improvement) and negative responses (unaltered or worsening) as far as tinnitus is concerned.

Of the 18 patients who had full graft take, 16 had positive tinnitus response and 2 had negative responses. Among the 5 patients who remained with tympanic membrane perforation in the postoperative, 3 had positive responses to tinnitus and 2 had negative responses. According to Fisher’s exact test, there was no significant association between graft take and tinnitus improvement (p=0.194).

## DISCUSSION

Tinnitus is considered an abnormal activity of the auditory pathway, usually interpreted as a sound by the central nervous system. The association between tinnitus and hearing loss happens in 85 to 96% of the cases^4^. Thus, conductive hearing loss takes on a fundamental importance, since it can be reverted by means of a middle ear surgery.

We noticed that the hearing loss discomfort was greater than that caused by tinnitus in all the time points considered. This agrees with our daily practice that shows that patients with chronic simple otitis media are always more unhappy with their hearing loss and/or otorrhea, and often times only mention tinnitus when questioned about it.

In our study, 82.6% of the patients had satisfactory responses as far as their tinnitus was concerned (remission or improvement) after tympanoplasty when preoperative audiograms showed only conductive hearing loss. Despite having planned our study to exclude the patients that developed sensorineural or mixed hearing loss in the postoperative (in order to exclude those tinnitus that could still remain because of this component), no patient had such development.

We did not include acuphenometry (tinnitus measurement) in our study, since according to Jastreboff (1990), the tinnitus-caused discomfort happens due to a limbic system and autonomic nervous system activation, not bearing association with acuphenometry results, since tinnitus usually happens in a frequency around that of the hearing loss and its intensity varies between 5 and 10dB HL4.

We did not find studies with similar methodology to the present one in the literature, since most of the tinnitus studies associated with middle ear surgery are retrospective in nature. Saito et al. (1999) reported tinnitus improvement in 50% of the patients by different middle ear diseases; of those, 22 are cases of chronic otitis media, five of chronic cholesteatomatous otitis media, two of ossicular chain malformations and one of otospongiosis5. Differently from this, Helms (1981) reported tinnitus remission after surgery in 1/3 of the patients, also remaining unaltered and getting worse in 1/3, respectively^6^.

Hearing loss, even with a primary conductive characteristic, causes a reduction in afferent stimuli of the central auditory pathways. Since the efferent pathways have modulating properties, hearing loss reduces its suppressive action because it is no longer necessary. The efferent pathways dysfunction is one of the possible mechanisms causing tinnitus^7^.

Tympanoplasties usually improve tonal thresholds^8^, ^9^, ^10^, ^11^, ^12^ and the favorable tinnitus results are very likely a consequence of this improvement, since the proper vibration of middle ear fluids re-establishes both afferent and efferent stimuli^13^. This is the classical example of the probable association between hearing improvement and tinnitus improvement that may also be seen in the clinical practice in the postoperative of patients with middle ear effusion, ossicular chain fixation or broken; or external acoustic meatus stenosis^13^.

Preoperative gap average (29.58 dB HL) reduced to 16.90 dB HL and 16.73 dB HL in 30 and 180 days of postoperative, respectively. McGrew et al. (2004) made a retrospective analysis of the audiograms of 214 patients submitted to tympanoplasties and observed that the preoperative gap average (34.1 dB HL) reduced to 16.4 dB HL in the postoperative. Despite being small, the postoperative results were similar. This very study showed that 76 (36%) patients enjoyed gap closure of up to 10dB HL and 74 patients (34%) had a final gap between 11 and 20dB HL11. In our investigation, we obtained higher rates in these results, with 61% and 22% in 30 and 180 days, respectively. This difference may be explained because the work of McGrew et al. was retrospective, and this prevented us from obtaining data in 83 patients.

Tinnitus improvement after hearing restoration can also be explained by the theory of central auditory pathways hypersensitivity after reduction in afference. According to Heller and Bergman (1953), of the 80 individuals with normal hearing who were placed in an anechoic chamber, 94% had tinnitus during their stay in the chamber14. The central nervous system compensates for the reduction of certain stimulus by increasing the sensitivity of the centers involved in this perception. Thus, afference reduction caused by the air-bone gap would cause an increase in the sensitivity of the centers involved in this perception. Thus, afference reduction caused by the air-bone gap would result in an increase in the sensitivity of cochlear nuclei by any stimulus, including the spontaneous activities of the auditory pathway, resulting in the perception of tinnitus.^16^

It is known that the best results in chronic otitis media surgeries happen especially in the hands of well-trained surgeons8. Nonetheless, we found surprisingly good postoperative results in our series, even with the procedures carried out by resident physicians. Complete gap closure or maintaining a maximum gap of 10 dB HL was reached in 61% of the patients in the postoperative, and there were no cases of worsening or profound hearing loss in this sample. Considering postoperative responses in 180 days, there was an improvement in tonal thresholds in all the frequencies, except in the 8 KHz frequency, showing that the results attained in the short term (30 days) were maintained.

Tympanic membrane healing (graft take) happened in 18 (78%) cases. Vartiainen (1998) compared the surgeries done to the ear because of chronic disease by preceptors and residents, observing that the patients operated upon by residents had lower audiometric gain and lower graft take rate when compared to those patients operated upon by preceptors (10 and 14 dB HL; 78% and 95%, respectively)^8^. Gerber et al. (2000) did a retrospective study of patients submitted to tympanoplasty with tragal cartilage pericondrium or temporal muscle fascia, observing improvements in tonal thresholds and gap, regardless of the type of graft used9. Fukuchi et al. (2006) show that graft take does not depend on age, perforation size, graft type, time of disease, among others, despite the low success rate of 65% of graft take, they associated it to the surgeon’s skills, since all the procedures were carried out by resident physicians^12^.

In relation to gap closure in the postoperative, we observed that most of the patients reported positive results in their tinnitus, without association with residual gap. One patient who had a gap between 21-30dB HL reported tinnitus worsening and, at the time of the medical visit at 30 days of postop still had perforation seen at the otoscopy and otorrhea, and this could explain the worsening in his tinnitus. Among the 3 patients with a gap <10dB HL who remained with their tinnitus unaltered, one still had the perforation in his tympanic membrane, the second had important anxiety (under daily use of tranquilizer), and the third still complained of hearing loss (worked in a confectioners place and was overweight). According to Sanchez and Bento (2000), tinnitus may present multiple factors involved in its genesis (otologic, metabolic, neurologic, cardiovascular, dental, psychological, etc.) that, may all occur in the same patient^15^. Therefore, the patients in whom the tinnitus persisted despite improvements in their hearing, could have other concomitant conditions to justify their clinical presentation, contributing as associated factors.

Thus, our data complete the scarce literature on tinnitus results after tympanoplasties and serve as support for otorhinolaryngologists when facing tinnitus patients with simple chronic otitis media and conductive hearing loss as associated causes, having seen that this symptom can also be satisfactorily controlled surgically.

## CONCLUSION

Patients with tinnitus and hearing loss are excellent candidates to tympanoplasty in order to control tinnitus by hearing improvement.
